# Academic Medicine Faculty Perceptions of Work-Life Balance Before and Since the COVID-19 Pandemic

**DOI:** 10.1001/jamanetworkopen.2021.13539

**Published:** 2021-06-15

**Authors:** Susan A. Matulevicius, Kimberly A. Kho, Joan Reisch, Helen Yin

**Affiliations:** 1 Office of Faculty Affairs, The University of Texas Southwestern, Dallas; 2 Department of Internal Medicine, The University of Texas Southwestern, Dallas; 3 Department of Obstetrics and Gynecology, The University of Texas Southwestern, Dallas; 4 Department of Population and Data Sciences, The University of Texas Southwestern, Dallas; 5 Department of Physiology, The University of Texas Southwestern, Dallas

## Abstract

**Importance:**

How the COVID-19 pandemic has affected academic medicine faculty's work-life balance is unknown.

**Objective:**

To assess the association of perceived work-life conflict with academic medicine faculty intention to leave, reducing employment to part time, or declining leadership opportunities before and since the COVID-19 pandemic.

**Design, Settings, and Participants:**

An anonymous online survey of medical, graduate, and health professions school faculty was conducted at a single large, urban academic medical center between September 1 and September 25, 2020.

**Main Outcomes and Measures:**

Self-assessed intention to leave, reducing employment to part time, or turning down leadership opportunities because of work-life conflict before and since the COVID-19 pandemic.

**Results:**

Of the 1186 of 3088 (38%) of faculty members who answered the survey, 649 (55%) were women and 682 (58%) were White individuals. Respondents were representative of the overall faculty demographic characteristics except for an overrepresentation of female faculty respondents and underrepresentation of Asian faculty respondents compared with all faculty (female faculty: 649 [55%] vs 1368 [44%]; Asian faculty: 259 [22%] vs 963 [31%]). After the start of the COVID-19 pandemic, faculty were more likely to consider leaving or reducing employment to part time compared with before the pandemic (leaving: 225 [23%] vs 133 [14%]; *P* < .001; reduce hours: 281 [29%] vs 206 [22%]; *P* < .001). Women were more likely than men to reduce employment to part time before the COVID-19 pandemic (153 [28%] vs 44 [12%]; *P* < .001) and to consider both leaving or reducing employment to part time since the COVID-19 pandemic (leaving: 154 [28%] vs 56 [15%]; *P* < .001; reduce employment: 215 [40%] vs 49 [13%]; *P* < .001). Faculty with children were more likely to consider leaving and reducing employment since the COVID-19 pandemic compared with before the pandemic (leaving: 159 [29%] vs 93 [17%]; *P* < .001; reduce employment: 213 [40%] vs 130 [24%]; *P* < .001). Women with children compared with women without children were also more likely to consider leaving since the COVID-19 pandemic than before (113 [35%] vs 39 [17%]; *P* < .001). Working parent faculty and women were more likely to decline leadership opportunities both before (faculty with children vs without children: 297 [32%] vs 84 [9%]; *P* < .001; women vs men: 206 [29%] vs 47 [13%]; *P* < .001) and since the COVID-19 pandemic (faculty with children vs faculty without children: 316 [34%] vs 93 [10 %]; *P* < .001; women vs men: 148 [28%] vs 51 [14%]; *P* < .001).

**Conclusions and Relevance:**

In this survey study, the perceived stressors associated with work-life integration were higher in women than men, were highest in women with children, and have been exacerbated by the COVID-19 pandemic. The association of both gender and parenting with increased perceived work-life stress may disproportionately decrease the long-term retention and promotion of junior and midcareer women faculty.

## Key Points

**Question:**

How is the COVID-19 pandemic associated with academic medicine faculty perceptions of work-life integration?

**Findings:**

In this survey of 1186 medical, graduate, and health professional school faculty, more faculty considered leaving since the COVID-19 pandemic than before. Faculty with children, particularly female faculty with children, were more likely to consider leaving since the pandemic.

**Meaning:**

These findings suggest that the stressors of integrating work and life are higher in female faculty than male faculty, highest in women with children, and may have been heightened by the COVID-19 pandemic.

## Teaser

This survey study assesses the relationship of perceived work-life conflict with academic medicine faculty intention to leave, reduce employment to part time, or decline leadership opportunities since the COVID-19 pandemic.

## Introduction

The COVID-19 pandemic has altered the ways we live and work with far-reaching impacts on all sectors of society. In the United States, 9.8 million jobs were lost between February and December 2020.^1^ This job loss has disproportionately affected women, who accounted for 46% of the prepandemic workforce but have experienced 54% of pandemic-related job losses.^2^ Structural inequalities further affect parents who have significantly increased their time spent on household and childcare duties by an additional 27 hours per week.^1^ This change has disproportionately affected mothers of young children, who have experienced a 4- to 5-fold decrease in work hours than working fathers since the pandemic.^3^

The COVID-19 pandemic has not spared the field of medicine, magnifying both the unique and universal stressors faced by physicians and medical scientists. Even before the pandemic, the US health care system had put a great deal of stress on health care workers through systems of high workload, high administrative burdens, inefficiency, emphasis on high productivity, and a culture of constant availability.^4,5^ Gender differences in pay parity, promotion, and work distribution have unevenly affected female physicians, leading more female physicians to reduce their working hours to part time or leave the field of medicine entirely.^6^ In academic medicine, women were already underrepresented in senior leadership positions before the pandemic. Although women make up 41% of all full-time academic medical school faculty, they account for 18% of academic chairs, 18% of deans, and 25% of full professors.^7^ The COVID-19 pandemic threatens to cause a regressive effect on the positive trends in gender equity and success in academic medicine unless action is taken.

To better understand how the COVID-19 pandemic is associated with faculty work-life conflict within our large urban academic medical institution, we conducted a campus-wide faculty survey to evaluate the perceived stress of the pandemic and maintaining work-life balance has affected faculty intention to leave, consideration of reducing their employment to part time and turning down leadership opportunities.

## Methods

This survey study was granted institutional review board exemption by the authority of the University of Texas Southwestern because analysis of the anonymous survey data was classified as nonhuman participant research. This study followed the American Association for Public Opinion Research (AAPOR) reporting guideline. Informed consent was not obtained because the study was of minimal risk, the survey was conducted anonymously and voluntarily, and the study involved no procedures for which written consent would normally be required outside of the research context.

### Sample

In September 2020, all faculty (n = 3088) at the University of Texas Southwestern in Dallas, Texas, were emailed an anonymous survey through their university-assigned email address inquiring about faculty perceptions of the effects of the COVID-19 pandemic on their career.^8^ The survey was conducted from September 1 to September 25, 2020. All active faculty at the time of the survey were emailed to participate based on university employment records, and the email survey invitation was sent from an account in the Office of Faculty Affairs, which is excluded from spam filters. The purpose of the survey was to assess the perceived effects of the COVID-19 pandemic on our faculty, with a particular focus on issues of work-life balance. Children were defined as any household member 18 years or younger to capture nonadult dependents as well as young adults (ie, 18-year-olds) who may be attending high school and living at home. Demographic data were collected on academic track, rank, department, gender, race, the presence of children at home, and the percentage of time on campus vs remote work. Data about the percentage of virtual vs in-person schooling for faculty children and who assisted their children with virtual learning was also collected for faculty with children. Faculty were also asked about the perceived association of COVID-19 with their degree of work-life balance.

### Measures

The survey asked faculty whether they had considered leaving the institution because of work-life balance issues before (March 2019 to March 2020) and since (March 2020 to September 2020) the COVID-19 pandemic with binary yes or no options. The survey asked faculty whether they had considered or were already working part time because of work-life balance issues before and since the COVID-19 pandemic with binary yes or no options. The survey asked faculty whether they had turned down opportunities for career advancement because of work-life balance issues before and since the COVID-19 pandemic with binary yes or no options.

### Statistical Analysis

Survey respondent demographic characteristics were compared with all faculty demographic characteristics. Faculty were compared by academic rank, track, gender, and presence or absence of children in their prevalence of intent to leave, reducing their employment to part time, or turning down leadership opportunities both before and since the pandemic. Differences across demographic characteristics were compared with the χ^2^ test. Differences between paired before and since the pandemic answers were compared using the McNemar test. The Bonferroni correction was applied to make a corrected *P* value of <.002 (ie, corrected *P* = .05/26) be required for results to be considered significant to account for the risk of type 1 error because of multiple comparisons. Statistical tests were 2-tailed and data analysis was generated using SAS software version 9.4 (SAS Institute).

## Results

Of the 1186 of 3088 faculty members (38%) who answered the survey, 649 (55%) were women, and 682 (58%) were White individuals. Two faculty members explicitly refused to participate. Both questions about before and since the COVID-19 pandemic were answered by 966 respondents (81%) for the intention to leave questions, 953 respondents (80%) for the considering or already working part time questions, and 928 respondents (78%) for the turning down leadership opportunities questions. Women and White faculty were more likely to respond to all 3 questions compared with nonrespondents (intent to leave: women, 552 [57%] vs 98 [45%]; White respondents, 572 [59%] vs 110 [50%]; reduce their employment to part time: women, 540 [57%] vs 110 [47%]; White respondents, 568 [60%] vs 114 [49%]; turn down leadership opportunities: women, 528 [57%] vs 122 [47%]; White respondents, 557 [60%] vs 125 [48%]); however, the respondents and nonrespondents were similar in terms of faculty rank and academic track (eTable in the Supplement). The overall survey respondents were representative of the overall faculty in terms of academic tracks, ranks, and departmental affiliation (basic science vs clinical). There was an overrepresentation of women (649 of survey respondents [55%] vs 1368 of 3088 faculty [44%]; *P* < .001) and underrepresentation of Asian individuals (259 [22%] vs 963 [31%] *P* < .001) (Table 1). Of our survey respondents, 652 (55%) reported having children 18 or younger. Among these faculty, 469 (72%) had children doing most of their schooling online. Of the faculty who responded, 363 (62%) were responsible for assisting their children with virtual learning either personally or in conjunction with their spouse in addition to their own professional roles.

**Table 1.** Characteristics of All Faculty Compared With Survey Respondents

Characteristic	Faculty, No. (%)	
	All faculty (n = 3088)	Survey respondents (n = 1186)
Gender		
Female	1368 (44)	649 (55)
Male	1740 (56)	475 (40)
Prefer not to say	0	60 (5)
Race/ethnicity		
Black/African American	124 (4)	30 (3)
Hispanic/Latinx	124 (4)	58 (5)
Asian	963 (31)	259 (22)
White	1678 (54)	682 (58)
Prefer not to say	218 (7)	157 (13)
Faculty rank		
Instructor	124 (4)	39 (3)
Assistant professor	1523 (49)	528 (45)
Associate professor	622 (20)	266 (22)
Professor	653 (21)	256 (22)
Faculty associate	124 (4)	45 (4)
Other or prefer not to say	62 (2)	52 (4)
Academic track		
Clinical scholar	280 (9)	113 (10)
Clinician educator	1927 (62)	702 (59)
Research	280 (9)	109 (9)
Accruing tenure	373 (12)	169 (14)
Other or prefer not to say	186 (6)	93 (8)
Department affiliation		
Basic science	404 (13)	167 (14)
Clinical	2642 (85)	960 (81)
School of health professions	62 (2)	37 (3)
Library	0	6 (0.5)
Prefer not to say	NA	16 (1)

Abbreviation: NA, not applicable.

### All Faculty

**Figure 1.**
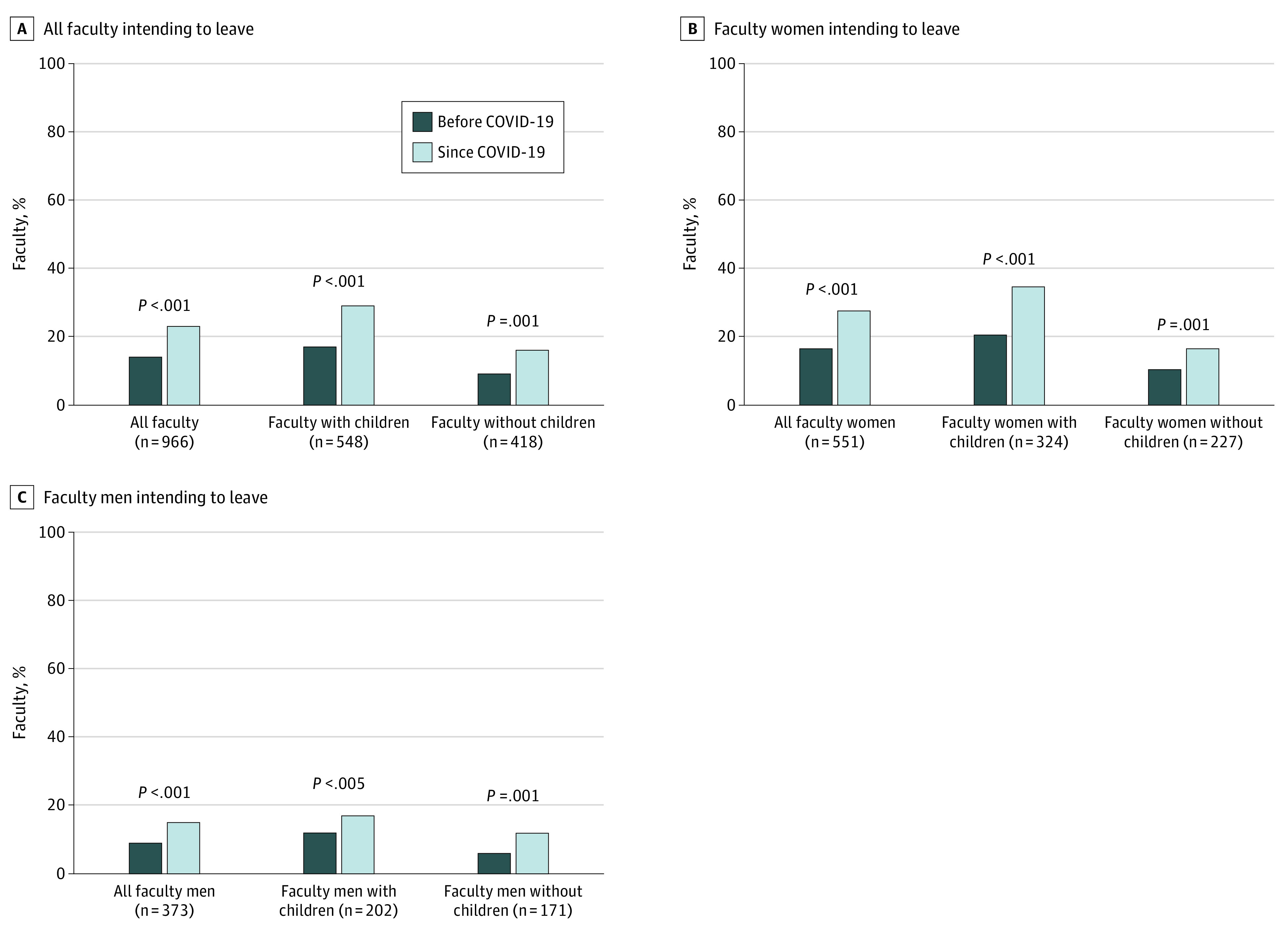
Intent to Leave the University of Texas Southwestern Before and Since COVID-19

**Figure 2.**
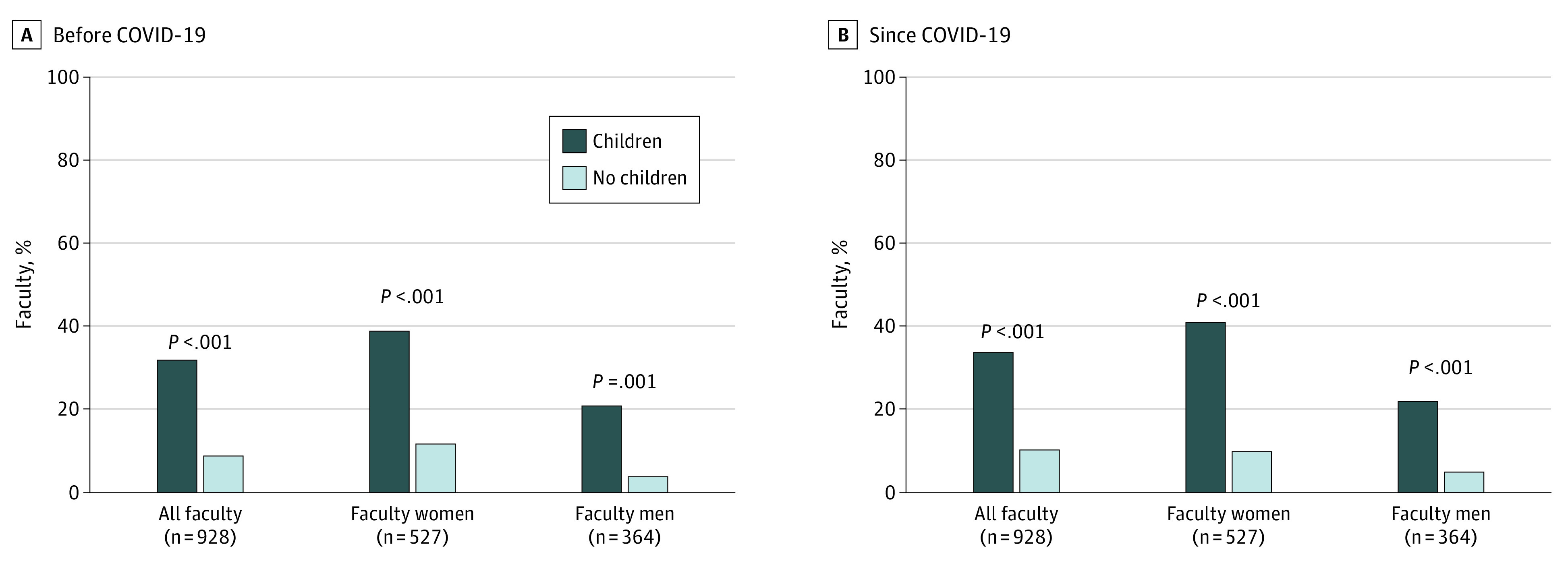
Turning Down Leadership Opportunities Before COVID-19 and Since COVID-19 Based on Gender and Status of Having Children Aged 18 Years or Younger

Compared with before the pandemic, more faculty considered leaving because of work-life balance and/or childcare stressors since the COVID-19 pandemic (133 [14%] vs 225 [23%]; *P* < .001) (Figure 1). Similarly, compared with before the pandemic, more faculty have considered reducing their employment to part time by continuing or transitioning to part-time work since the COVID-19 pandemic (206 [22%] vs 281 [29%]; *P* < .001) (Table 2). Faculty of all academic tracks reported increased occupational stress, including research and tenure-accruing faculty. Assistant professors reported being the most affected by these stressors (Table 2 and Table 3). Since the COVID-19 pandemic, 462 (39%) of faculty reported decreased research productivity, 344 (29%) delayed manuscript submissions, 273 (23%) had declined teaching or speaking opportunities, and 261 (22%) increased their clinical workload with a perceived detrimental effect on their academic productivity. There was no statistically significant difference in any of these elements by gender. These trends were consistent across academic tracks and ranks. There was no difference before and since the COVID-19 pandemic in the overall faculty turning down leadership opportunities because of work-life balance issues (204 [22%] vs 213 [23%]; *P* = .45) (Figure 2).

**Table 2.** Faculty Considering Working Part Time Because of Work-Life Balance Issues

Academic track	Respondents, No. (%)	Respondents considering part-time work or already working part time, No. (%)		*P* value
		Before COVID-19	Since COVID-19	
All faculty	953	206 (22)	281 (29)	<.001
Clinical scholar	94	17 (18)	21 (22)	.31
Clinician educator	574	161 (28)	210 (37)	<.001
Research	87	10 (11)	19 (22)	.04
Tenured or accruing tenure	136	5 (4)	15 (11)	.01
Faculty Rank				
Instructor	32	5 (16)	5 (16)	1.00
Professor	855			
Assistant	419	96 (23)	149 (36)	<.001
Associate	222	55 (25)	75 (34)	.04
Full	214	35 (16)	39 (18)	.61
Gender				
Female	539	153 (28)	215 (40)	<.001
Male	372	44 (12)	49 (13)	.45
With children				
All faculty	535	130 (24)	213 (40)	<.001
Female	318	111 (35)	173 (54)	<.001
Male	196	15 (8)	29 (15)	.003
Without children				
All faculty	418	76 (18)	68 (16)	.28
Female	221	42 (19)	42 (19)	1.0
Male	176	29 (16)	20 (11)	.005

**Table 3.** Consideration of Leaving the University of Texas Southwestern Because of Work-Life Balance Issues

Academic track or rank	Respondents, No. (%)	Faculty, No. (%)		*P* value
		Before COVID-19	Since COVID-19	
All faculty	966	133 (14)	225 (23)	<.001
Clinical scholar	92	15 (17)	22 (24)	.05
Clinician educator	584	100 (17)	159 (27)	<.001
Research	89	7 (8)	19 (21)	<.001
Tenured or accruing tenure	140	4 (3)	13 (9)	.007
Faculty rank				
Instructor	31	2 (6)	6 (19)	.05
Assistant	426	76 (18)	126 (30)	<.001
Associate	222	34 (15)	58 (26)	.001
Full	219	15 (7)	20 (9)	.20
Gender				
Female	551	94 (17)	154 (28)	<.001
Male	373	34 (9)	56 (15)	<.001
With children				
All faculty	548	93 (17)	159 (29)	<.001
Female	324	68 (21)	113 (35)	<.001
Male	202	24 (12)	34 (17)	.050
Without children				
All faculty	418	38 (9)	67 (16)	<.001
Female	227	25 (11)	39 (17)	.010
Male	171	10 (6)	21 (12)	<.001

### Gender

Faculty women were nearly twice as likely to have considered leaving since the COVID-19 pandemic compared with before the pandemic (154 [28%] vs 94 [17%]; *P* < .001) (Figure 1). Faculty women were nearly 3 times as likely to have considered or were already working part time both before and since the COVID-19 pandemic compared with men (before: 176 [30%] vs 50 [13%]; *P* < .001; since: 215 [40%] vs 49 [13%]; *P* < .001). Similarly, faculty women were more likely than men to turn down leadership opportunities because work-life balance issues before and since the COVID-19 pandemic (before: 159 [28%] vs 48 [13%]; *P* < .001; since: 150 [28%] vs 54 [14%]; *P* < .001).

### Working Faculty Parents 

Working faculty parents compared with faculty without children were not statistically more likely to consider leaving before the COVID-19 pandemic but were statistically significantly more likely to consider leaving since the COVID-19 pandemic (before: 93 [17%] vs 38 [9%]; *P* = .002; since: 159 [29%] vs 67 [16%]; *P* < .001) (Figure 1). Similarly, working parents were 1.6 times more likely to consider or have already decreased their work to part time since the COVID-19 pandemic compared with before the pandemic (214 [40%] vs 128 [24%]; *P* < .001) (Table 2). Irrespective of before or since the pandemic, working parents were 3 times more likely to turn down leadership opportunities because of childcare or work-life balance issues compared with faculty without children (before: 166 [32%] vs 37 [9%]; *P* < .001; since COVID-19: 177 [34%] vs 40 [10%]; *P* < .001) (Figure 2).

Faculty women with children compared with faculty women without children were not statistically more likely to consider leaving before the pandemic but were statistically significantly more likely to consider leaving since COVID-19 (before: 68 [21%] vs 25 [11%]; *P* = .002; since: 113 [35%] vs 39 [17%]; *P* < .001) (Figure 1). Similarly, faculty women with children were more likely to have considered being, or already were, working part time both before and since COVID-19 (before: 111 [35%] vs 42 [19%]; *P* < .001; since: 172 [54%] vs 42 [19%]; *P* < .001) (Table 2). They were also more likely to have turned down leadership opportunities because of work-life conflict both before and since COVID-19 (before: 120 [39%] vs 26 [12%]; *P* < .001; since: 127 [41%] vs 21 [10%]; *P* < .001) compared with faculty women without children (Figure 2).

Faculty men with children compared with faculty men without children were not statistically significantly more likely to consider leaving before or since COVID-19 (before: 24 [12%] vs 10 [6%]; *P* = .03; since: 34 [17%] vs 21 [12%]; *P* = .21) (Figure 1). There was no statistically significant difference between faculty men with children compared with men without children in considering or already working part time before or since the COVID-19 pandemic (15 [8%] vs 29 [16%]; *P* = .009) or since COVID-19 (29 [15%] vs 20 [11%]; *P* = .33) (Table 2). However, faculty men with children were more likely to have turned down leadership opportunities because of work-life conflict both before and since COVID-19 (before: 41 [21%] vs 7 [4%]; *P* < .001; since: 43 [22%] vs 9 [5%]; *P* < .001).

## Discussion

The findings of our study of 1186 faculty at a large, urban academic medical institution suggest that the COVID-19 pandemic has been a major stressor for most faculty, as illustrated by an increased prevalence of intent to leave or consideration of reducing their employment to part time since the pandemic began. Working parents, regardless of gender, were more likely to encounter work-life integration issues both before and since the COVID-19 pandemic. Women were more likely than men to report being affected by these stressors, with women with children 18 years or younger reporting the greatest impact. Faculty women with children were the group most likely to report work-life balance stress even before the pandemic, and the pandemic heightened this further. This association of both gender and parenting with increased perceived stress may disproportionately decrease the long-term retention and promotion of junior and midcareer women faculty.

Our study showed an increased rate of intention to leave the University of Texas Southwestern since the pandemic in all faculty surveyed, in parents of children aged 18 years or younger, and with the highest rate in women with children. Our results corroborate the findings of a 2021 study^9^ from the University of Utah that showed that faculty and trainees with caregiving roles were more likely than those without caregiving roles to consider leaving the workforce or reducing their hours since the pandemic. In our study, faculty who were mothers were more likely to consider leaving or already had or were considering reducing their employment to part time both before and since the pandemic compared with faculty women without children, highlighting the universal stress of caregiving independent of the pandemic. Prior research suggests that academic faculty at a large academic medical center who intended to leave within 2 years had a 3-fold higher rate of actually leaving than those who did not.^10^ Furthermore, expressing an intention to leave is highly associated with burnout.^10,11^ In academic settings, burnout is linked to decreased academic productivity, loss of midcareer mentorship, and economic loss associated with physician turnover and loss of patient-doctor relationship continuity.^12,13^ In our study, 72% of faculty parents had children who attended school virtually more than 50% of the time. Since the pandemic, academic faculty with children aged 5 years or younger reported having completed fewer peer-review assignments, attended fewer funding panel meetings and had fewer first-author manuscript submissions than before the pandemic.^14^ Academic mothers in science submitted fewer manuscripts, registered fewer new projects, and had lower publication rates than before the COVID-19 pandemic.^15,16^ This loss in academic productivity has implications for promotion and the pipeline for leadership in academic medicine unless concerted efforts are made to account for or reverse this trend.

Faculty women in our study were more likely than faculty men to turn down leadership opportunities because of work-life conflict both before and since the COVID-19 pandemic. Work-life conflict and role strain are significantly associated with decreased leadership-seeking behaviors for academic women and contribute to the gender inequities we see in academic promotion and leadership.^17^ Women faculty spend more time on internal service activities (eg, departmental, institutional) than men, who spend more of their time on national service activities (eg, editorial boards, professional societies), thereby enhancing the national reputation and career advancement of male faculty.^18^ This excess burden of local service activities strains women’s ability to accept additional leadership opportunities and diminishes the pool of faculty in the leadership pipeline.

Part-time faculty appointments can further increase the gender gap. In our study, women were 3 times more likely than men to consider or already be employed part time both before and since the pandemic. Part-time faculty perceive that they perform more unpaid work, have fewer research opportunities, a slower career trajectory, and may be less likely to take on leadership appointments.^19,20^ Without true change in the culture of medicine to support work-life integration and family-friendly work policies, further disillusionment in academic careers may occur and threaten the future of academic medicine as an institution.^21^

### Limitations

This study has limitations. The survey did not specify why faculty members were considering leaving (ie, desire to leave this specific academic institution, leave academic medicine, or leave the practice of medicine or research). As an anonymous, voluntary survey, we did not have individual respondent data about the length of employment or retirement benefits and cannot correct for potential confounding effects of incentives or generational effects on intention to leave or reduce their employment to part time. We did not differentiate between faculty already working part time vs those considering working part time. We did not identify marital status or spousal employment status and, therefore, cannot assess the differential impact of the COVID-19 pandemic based on these factors. We relied on retrospective self-assessment of perceived stress before and since the pandemic. This could lead to recall bias, thereby accentuating or diminishing the relative magnitude of difference between their perceptions of stress before vs since the COVID-19 pandemic. Not all respondents to the survey answered both the before and since the COVID-19 pandemic questions. Women and White faculty were more likely to respond to all 3 questions (intent to leave, reduce their employment to part time, or turn down leadership opportunities) compared with nonrespondents; however, the respondents and nonrespondents were similar in terms of faculty rank and academic track (eTable in the Supplement). The response rate of 38% may lead to sampling bias, however our study respondents were similar in the distribution of rank, track, and departmental affiliation. Multiple comparisons were performed in this survey, risking statistically significant differences based on random sampling error alone.

## Conclusions

In this survey study, academic medical faculty, particularly working parents and especially working mothers, reported that since the COVID-19 pandemic, they were considering leaving their jobs, reducing their employment to part time, or turning down leadership roles. Better support of working parents, specifically working mothers, through flexible work policies, improved childcare and parental leave programs, more equitable sharing of unpaid care hours between women and men, and active acknowledgment of the effects of work-life conflict on academic productivity and fulfillment are paramount to ensuring academic medicine does not lose talented faculty and proactively combats gender inequity and gender-based advancement regression.

## Notes

Supplement. **eTable.** Demographic Comparison of Responders vs Nonresponders for Intention to Leave, Consideration or Already Part-time, and Turning Down Leadership Opportunities
