# Supplementary material for: Academic Medicine Faculty Perceptions of Work-Life Balance Before and Since the COVID-19 Pandemic
Source: JAMA Netw Open. 2021 Jun 15;4(6):e2113539. doi: 10.1001/jamanetworkopen.2021.13539 (PMC8207238; doi:10.1001/jamanetworkopen.2021.13539)
Supplement: Supplement. — eTable. Demographic Comparison of Responders vs Nonresponders for Intention to Leave, Consideration or Already Part-time, and Turning Down Leadership Opportunities [file jamanetwopen-e2113539-s001.pdf]

Supplemental Online Content

Matulevicius SA, Kho KA, Reisch J, Yin H. Academic medicine faculty perceptions of work-life balance before and since the COVID-19 pandemic. *JAMA Netw Open*. 2021;(4)6:e2113539. doi:10.1001/jamanetworkopen.2021.13539

eTable. Demographic Comparison of Responders vs Nonresponders for Intention to Leave, Consideration or Already Part-Time, and Turning Down Leadership Opportunities

This supplemental material has been provided by the authors to give readers additional information about their work.

© 2021 Matulevicius SA et al. *JAMA Network Open.*

eTable 1. Demographic Comparison of Responders vs Nonresponders for Intention to Leave, Consideration or Already Part-Time, and Turning Down Leadership Opportunities

	**Intent to Leave**		**Decreased Professional Effort**		**Turn Down Leadership**	
**Respondent Category No. (%)**	**Respondents** (n=966)	**Non-Respondents** (n=220)	**Respondents** (n=953)	**Nonrespondents** (n=233)	**Respondents** (n=928)	**Nonrespondents** (n=258)
**Gender**						
Female	552 (57)	98 (45)	540 (57)	110 (47)	528 (57)	122 (47)
Male	373 (31)	102 (47)	372 (39)	103 (44)	364 (39)	111 (43)
Prefer Not to Say	41 (4)	20 (9)	41 (4)	19 (8)	36 (4)	24 (9)
**Ethnicity**						
Black/African American	24 (2)	6 (3)	23 (2)	7 (3)	21 (2)	9 (3)
Hispanic/LatinX	49 (5)	9 (4)	47 (5)	11(5)	47 (5)	11 (4)
Asian	213 (22)	47 (21)	212 (22)	48 (21)	204 (22)	56 (22)
White	572 (59)	110 (50)	568 (60)	114 (49)	557 (60)	125 (48)
Prefer Not to Say	108 (11)	48 (31)	103 (11)	53 (23)	99 (11)	57 (22)
**Faculty Rank**						
Instructor	31 (3)	8 (4)	31 (3)	8 (3)	30 (3)	9 (3)
Assistant Professor	426 (44)	103 (47)	419 (44)	110 (47)	404 (44)	125 (48)
Associate Professor	222 (23)	44 (20)	222 (19)	44(19)	218 (23)	48 (19)
Professor	219 (23)	37 (17)	214 (22)	42 (18)	214 (23)	42 (16)
Faculty Associate	29 (3)	16 (7)	29 (3)	16 (7)	30 (3)	15 (6)
Other/Prefer Not to Say	39 (4)	12 (5)	38 (4)	13 (6)	32 (3)	19 (7)
**Academic Track**						
Clinical Scholar	92 (9)	21 (10)	94 (10)	19 (17)	92 (10)	21 (8)
Clinician Educator	584 (60)	119 (54)	574 (60)	129 (55)	562 (61)	141 (55)
Research	89 (9)	20 (9)	87 (9)	22 (9)	82 (9)	27 (10)
Tenure-Accruing	140 (14)	29 (12)	136 (14)	33 (14)	136 (15)	33 (13)
Other/Prefer Not to Say	61 (6)	31 (14)	62 (7)	30 (33)	56 (6)	36 (14)

© 2021 Matulevicius SA et al. *JAMA Network Open.*
